# Do citizens have minimum medical knowledge? A survey

**DOI:** 10.1186/1741-7015-5-14

**Published:** 2007-05-31

**Authors:** Lucas M Bachmann, Florian S Gutzwiller, Milo A Puhan, Johann Steurer, Claudia Steurer-Stey, Gerd Gigerenzer

**Affiliations:** 1 Horten Centre for patient oriented research, University of Zurich, Bolleystrasse 40, Postfach Nord, CH-8091 Zurich, Switzerland; 2 Medical Policlinic, Department of Internal Medicine, University Hospital, Zurich, Switzerland; 3 Max Planck Institute for Human Development, Center for Adaptive Behavior & Cognition, Berlin, Germany

## Abstract

**Background:**

Experts defined a "minimum medical knowledge" (MMK) that people need for understanding typical signs and/or risk factors of four relevant clinical conditions: myocardial infarction, stroke, chronic obstructive pulmonary disease and HIV/AIDS. We tested to what degree Swiss adult citizens satisfy this criterion for MMK and whether people with medical experience have acquired better knowledge than those without.

**Methods:**

Questionnaire interview in a Swiss urban area with 185 Swiss citizens (median age 29 years, interquartile range 23 to 49, 52% male). We obtained context information on age, gender, highest educational level, (para)medical background and specific health experience with one of the conditions in the social surrounding. We calculated the proportion of MMK and examined whether citizens with medical background (personal or professional) would perform better compared to other groups.

**Results:**

No single citizen reached the full MMK (100%). The mean MMK was as low as 32% and the range was 0 -72%. Surprisingly, multivariable analysis showed that participants with a university degree (n = 84; β (95% CI) +3.7% MMK (0.4–7.1) p = 0.03), (para)medical background (n = 34; +6.2% MMK (2.0–10.4), p = 0.004) and personal illness experience (n = 96; +4.9% MMK (1.5–8.2), p = 0.004) had only a moderately higher MMK than those without, while age and sex had no effect on the level of MMK. Interaction between university degree and clinical experience (personal or professional) showed no effect suggesting that higher education lacks synergistic effect.

**Conclusion:**

This sample of Swiss citizens did not know more than a third of the MMK. We found little difference within groups with medical experience (personal or professional), suggesting that there is a consistent and dramatic lack of knowledge in the general public about the typical signs and risk factors of relevant clinical conditions.

## Background

Lay persons cannot know everything about an illness, even about frequent illnesses. But there are two things that he or she should know: characteristic symptoms, and risk factors that are under their control. Particularly for serious acute conditions such as myocardial infarction or stroke, it could be vital to know the early symptoms and signs because immediate medical care is essential to favourably influence the course of illness. If we assume that only 50% of the general public know about early symptoms, it is clear that direct costs and also costs related to mortality and morbidity as a result of lack of knowledge would be substantial. Moreover, from a preventative medical point of view, limited insight into mechanisms leading to a serious condition could lead to unconscious risk behaviour.

Commonly, in the context of health literacy, research has focused on an individual's ability "to obtain, process, and understand basic information and services needed to make appropriate health decisions" [1]. However, to date, little has been known about the level of health knowledge in the general public. Some studies have investigated the relationship between attitudes and health behaviour [2-4], focused on one single health issue [3,5,6] or did not investigate the general public [4,7].

In this study, we defined a minimum medical knowledge (MMK) for different conditions on a scale that can measure individual knowledge between 0–100%. The scale can be applied broadly. We assumed that people with personal experience or professional medical training would reach the maximum MMK more often than those who do not.

The reason for this hypothesis is the assumption that people with some (para)medical background (professional experience) or those confronted (directly as a patient or indirectly as e.g. a relative of a patient) with one of the conditions will have acquired more knowledge than those with no professional or personal experience. We also expected that a higher education would have a synergistic effect on MMK when combined with personal experience. The reason for this hypothesis is the assumption that people do not actively search for disease-specific information unless they are exposed to a specific condition, and that people with a higher education might be more skilled in gathering such information.

To test these hypotheses, we interviewed citizens of an urban area in Switzerland and assessed their knowledge on typical signs and/or risk factors for four clinical conditions that have a significant impact on the healthcare system: myocardial infarction, stroke, chronic obstructive pulmonary disease and HIV/AIDS.

## Methods

### Questionnaire development

We contacted twelve clinical experts (three experts per specialty) to define the minimum knowledge an average citizen should have in relation to risks and symptoms of four illnesses with major impact on health status and economics: chronic obstructive pulmonary disease (COPD), HIV infection, heart attack and stroke (experts names and specialities are given in Additional file 1). Experts were asked to be careful to state just the most common set of symptoms that should be known by everyone, excluding uncommon factors or unusual presentation of symptoms. Experts were presumed to know much more about the conditions than the 100% "minimum knowledge" level. Additional file 2 defines what we considered minimum knowledge.

We transformed their statements into two (three for HIV) questions per illness and developed a questionnaire that could be completed within five minutes. For each question we defined a minimal set of correct answers (see Additional file 2). We tested the questionnaire on 38 subjects to obtain the final dataset. We pre-printed the questionnaires to optimise the course of the interview. The cantonal ethical committee of Zurich stated that this study did not require ethical approval.

### Interviewer

Six interviewers received an oral and written instruction on how to conduct the interviews. They were trained on 38 subjects.

### Participants

We recruited participants at six busy locations in Zurich, Switzerland. Eligible participants were randomly approached and asked whether they would agree to take part in the study. We approached 272 pedestrians, and 185 (68%) were willing to take part. Mean age (SD) was 37 (18) and 97/185 (52%) were male. Table 1 has a detailed description of the study participants. We immediately interviewed all German-speaking individuals after they gave verbal consent. From each participant, we obtained information on age, gender, highest educational level, (para)medical background and specific personal experience with one of the conditions in the social surrounding. Each question was put to the participant and the corresponding replies recorded. We offered no incentives for study participation.

### Statistical analysis

First, the cumulative number of correct replies was counted, and for simplicity we calculated the correct MMK proportion (correct replies/MMK; Additional file 2) across all questions. Assessment of correct replies was performed in duplicate according to the predefined replies, and discordances between the assessors were discussed. In cases of continued disagreement, particularly if the participant used an unusual term, we classified the answer as correct. There are nine questions, where the minimal correct answer varies between one and five responses. The total number of correct answers is 25. The MMK level for a person is therefore the total number of correct replies divided by 25 × 100, to give a percentage score. Secondly, we examined the influence of age (continuous data), gender (female/male), highest educational level (university or other), (para)medical background (yes/no) and specific personal experience with one of the conditions in the social surrounding (yes/no) as independent variables, and the cumulative proportion of correct replies as the dependent variable using a linear multivariable regression model. Then we examined the interaction between university degree and (para)medical background, university degree and personal experience with one of the conditions and (para)medical background, and experience with one of the conditions using interaction terms along with the terms of the main effects. Finally, we investigated whether people with personal experience of one of the conditions would have a higher MMK for the corresponding questions. For this purpose we calculated the MMK for the corresponding condition. Analysis was performed using the Stata 9.2 statistical software package (StataCorp, 4905 Lakeway Drive, College Station, TX, USA).

## Results

No single citizen reached the full MMK (100%). The mean proportion (95% CI) of MMK was 32% (26 to 37); the range was 0–72%. Multivariable analysis showed that participants with a university degree (n = 84; β (95% CI) +3.7% MMK (0.4 to 7.1), p = 0.03), (para)medical background (n = 34; +6.2% MMK (2.0 to 10.4), p = 0.004) and personal illness experience (n = 96; +4.9% MMK (1.5 to 8.2), p = 0.004) had only a moderately higher MMK than those without. Age (-0.05% MMK (-0.2 to 0.04), p = 0.25) and male sex (-1.3% MMK (-4.7 to 2.1), p = 0.45) had no effect on results. The assessment of interactions between university degree and personal experience with a condition (p = 0.16), university degree and (para)medical background (p = 0.26) and personal experience with a condition and (para)medical background (p = 0.74) showed no effect on the MMK, suggesting that higher education has no synergistic effect. Hence, the mean MMK of, for example, a paramedic with a university degree and personal illness experience would be 47%.

For the conditions of stroke and heart attack, we had a sufficiently high number of respondents with a personal experience in their social surroundings (stroke n = 50 (27%); heart attack n = 59 (32%)) to assess the difference in the MMK compared to respondent without such experience. For questions on stroke, the subgroup with personal experience had a markedly higher MMK (+9.3% (4.3 to 14.4), p < 0.001) whereas for questions in relation to heart attack that difference was only small (+2.5% (-1.0 to 6.0), p = 0.16). For the other two conditions, HIV/AIDS and COPD, the number of participants with personal experience was too small for subgroup analyses (HIV/AIDS, n= 6; COPD, n = 12). Participants with para(medical) backgrounds gave more replies than others (β (95% CI)) +2.7 (0.8 to 4.6), p = 0.007).

## Discussion

In this survey among Swiss citizens, we found a considerable level of ignorance in relation to the symptoms of and risks for frequently found and important illnesses. Contrary to our expectations, the two groups who were personally confronted with a specific disease or professionally confronted with patients' diseases showed only marginally more specific knowledge than the general population. Participants with experience of one of the illnesses tended to score somewhat higher than those without. However, this effect was moderate and inconsistent.

Compared to countries which offer free access to health care to the entire population as a right of citizenship regardless of the ability to pay, the Swiss health system, which is based on franchise and percentage share, is seen as one that encourages citizens to seek out and put into practice health information to avoid costs. We expected citizens to be well informed and have a minimum knowledge in relation to conditions with a substantial health burden. Nevertheless, on average only one third of MMK could be recalled in our sample. It is plausible to assume that the performance in other populations, particularly in those with social health systems, might be even lower.

To date, little has been known about the level of health knowledge in the general public. Among the few exceptions, we found one article that re-examined epidemics such as that of HIV/AIDS [8,9] and reported results that are in line with our findings. In the 2000 Progress of Nations Report the authors describe a disproportionately high incidence of HIV/AIDS among sub-Saharan African teachers [8]. The high death rate of teachers shows clearly that general literacy and health literacy do not necessarily go hand in hand [10].

Similarly, a majority of health counsellors advising low-risk heterosexual men who take an HIV test made inconsistent or erroneous statements about the interpretation of a positive test result [11].

This survey was not without its limitations. Our assessment was based on a convenience sample of limited size. Compared to the Swiss population as a whole, our participants had a higher education than average and tended to be younger. The participation rate of 68% might have caused further selection, although we think that our respondents were more likely to score higher than average, leading to an overestimation of knowledge. We cannot rule out that some Swiss particularities impede broad generalisation of our findings to other countries. Finally, our questionnaire was designed as a recall test, which is usually more challenging then a recognition test such as, for example, a multiple-choice exam.

Further research should aim at replicating our findings. More specifically, we think that a broad international comparison, with a focus on the relationship with different healthcare systems and perhaps also in respect to people's ideas about healthcare, is warranted.

In relation to practice one important question arises. How can we explain that university degree, (para)medical training or experience with one of the health conditions does not markedly increase health knowledge? We hypothesise that health is still not coupled with knowledge. It seems to be that the majority of people do not inform themselves, but follow social heuristics such as advice taking, imitation and authority (if he/she wears a white coat, trust him/her). Even economists, who are the proponents of rational thought, seem not follow their own advice but trust others when it comes to medical decisions [12]. In a survey, 133 economists were asked what they knew about the prostate specific antigen (PSA) test and whether or not they would undergo testing. Although only 5% of participants knew about the PSA test, two-thirds admitted not to weighing up the pros and cons of testing. The majority said that they follow their physician's advice and 10% stated that they would follow the advice of their spouse.

## Conclusion

In this sample, Swiss citizens did not know more than a third of MMK. We found little improvement from this low level within groups with medical experience (personal or professional), suggesting that there is a consistent and dramatic lack of knowledge in the general public about the typical signs of and risk factors for important clinical conditions.

## Competing interests

The author(s) declare that they have no competing interests.

## Authors' contributions

GG, LMB and JS initiated the study. FSG, LMB and MAP developed the protocol, CSS gave critical input. FSG coordinated data collection, LMB performed the analysis. GG and LMB drafted the manuscript, all authors provided intellectual input.

## Pre-publication history

The pre-publication history for this paper can be accessed here:



## Supplementary Material

Additional file 1 List of experts consulted for questionnaire development. Click here for file

Additional file 2 Questions and corresponding correct answers. Click here for file

## Figures and Tables

**Table 1:** Characteristics of the study population.

Number of participants (%)	185 (100%)
Sex	
Women	83 (44.9)
Men	97 (52.4)
Missing	5 (2.7)
	
Age	
< 20	7 (3.8)
20–34	101 (54.5)
35–49	34 (18.4)
50–64	24 (13)
65–79	14 (7.6)
80–94	5 (2.7)
Mean (median) age	36.8 (29)
Age range	17–88
	
Education	
Mandatory school	8 (4.3)
Secondary school	4 (2.2)
University entrance diploma	70 (37.8)
Apprenticeship	34 (18.4)
Vocational school	16 (8.6)
University	86 (46.5)
Advanced technical college	11 (5.9)
No answer	1 (0.5)
	
Medical education, self reported	
Yes	34 (18.4)
No	150 (81.1)
Missing	1 (0.5)
	
Medical education, specified	
Lab technician	2 (1.1)
Nurse	2 (1.1)
Physician	4 (2.2)
Medical secretary	1 (0.5)
Medical professorship	3 (1.6)
Veterinarian	2 (1.1)
Nurse's assistant	4 (2.2)
Research	3 (1.6)
Physiotherapist	1 (0.5)
Army/civil defence	3 (1.6)
First aid course	3 (1.6)
Miscellaneous	6 (3.2)
	
Affected by one or more conditions questioned	
Yes	99 (53.5)
No	85 (46)
Missing	1 (0.5)
	
Which condition?	
Stroke	50 (27)
COPD	12 (6.5)
HIV	6 (3.2)
Heart attack	59 (31.9)
Persons/close relatives affected by more than one condition	25 (13.5)
No answer	2 (1.1)

**Table 2:** Raw MMK results (number of participants (n), mean MMK value, Standard Deviation SD) broken down by male gender, university degree, (para)medical education, personal illness experience and by the overall sample.

**Subgroups**	**n (%)**	**mean MMK (SD)**
Male	97 (52)	33.6 (12.6)
University degree	86 (46)	36.4 (11.6)
(Para)medical education	34 (18)	39.5 (13.6)
Personal illness experience	96 (52)	36.4 (11.5)
		
Overall sample	185 (100)	34.2 (11.8)
