# Supplementary material for: Do citizens have minimum medical knowledge? A survey
Source: BMC Med. 2007 May 31;5:14. doi: 10.1186/1741-7015-5-14 (PMC1894984; doi:10.1186/1741-7015-5-14)
Supplement: Additional file 1 — List of experts consulted for questionnaire development. [file 1741-7015-5-14-S1.doc]

## Additional file 1 - List of experts consulted for questionnaire development

**COPD:**

Otto Brändli, M.D., Höhenklinik Wald, Zurich

Claudia Steurer-Stey, M.D., Outpatient Department of Internal Medicine, University Hospital, Zurich

Daniel Ritscher, M.D., Lungenpraxis Morgental, Zurich

**HIV:**

Prof. Manuel Battegay, M.D., Division of Infectious Diseases, University Hospital Basel

Prof. Rainer Weber, M.D., Division of Infectious Diseases and Hospital Hygiene, University Hospital, Zurich

Markus Flepp, M.D., Klinik Im Park, Zurich

**Heart attack:**

Prof. Thomas Lüscher, M.D., Head of Cardiology Division, University Hospital, Zurich

Prof. Osmund Bertel, M.D., Head of Cardiology Division, Stadtspital Triemli, Zurich

Ulrich Steiger, M.D., Zürich

**Stroke:**

Prof. Ralf Baumgartner, M.D., Department of Neurology. University Hospital Zurich

Prof. Heinrich Mattle, M.D., Department of Neurology, Inselspital, Berne

Prof. Jürg Kesselring, M.D., Department of Neurorehabilitation, Rehabilitation Center Valens
