# Supplementary material for: Do citizens have minimum medical knowledge? A survey
Source: BMC Med. 2007 May 31;5:14. doi: 10.1186/1741-7015-5-14 (PMC1894984; doi:10.1186/1741-7015-5-14)
Supplement: Additional file 2 — Questions and corresponding correct answers. [file 1741-7015-5-14-S2.doc]

## Additional file 2 - Questions and corresponding correct answers

| **Question 1** | **What risk factors are mainly responsible for developing COPD?** |
| --- | --- |
| Correct answers: | Smoking, environmental and genetic factors |
|  |  |
| **Question 2** | **What are the symptoms of COPD?** |
| Correct answers: | Cough, expectoration (sputum) |
|  |  |
| **Question 3** | **Do you know the symptoms of a stroke?** |
| Correct answers: | Palsy, speech disorder |
|  |  |
| **Question 4** | **What diseases, habits and life circumstances increase, according to your opinion, the risk of having a stroke?** |
| Correct answers: | Smoking, high blood pressure, abnormal blood fat, diabetes, [cardiac](http://dict.leo.org/ende?lp=ende&p=/gQPU.&search=cardiac) [arrhythmia](http://dict.leo.org/ende?lp=ende&p=/gQPU.&search=arrhythmia) |
|  |  |
| **Question 5** | **Is there a difference between HIV and AIDS?** |
| Correct answer: | Yes |
|  |  |
| **Question 6** | **How can one protect oneself from HIV infection?** |
| Correct answers: | Condom, sexual abstinence, no direct contact with body fluids |
|  |  |
| **Question 7** | **Is HIV infection curable?** |
| Correct answer: | No |
|  |  |
| **Question 8** | **What can be symptoms of a heart attack?** |
| Correct answers: | Chest pain, radiating pain, unconsciousness |
|  |  |
| **Question 9** | **What diseases, habits and life circumstances increase, according to your opinion, the risk of having a heart attack?** |
| Correct answers: | Smoking, high blood pressure, abnormal blood fat, diabetes, genetic factors |

The MMK is computed in the following way. There are nine questions where the minimal correct answer varies between one and five responses. The total number of possible correct answers is 25. The MMK of a person is the total number of correct replies divided by 25, multiplied by 100 to give a percentage score. Please note that MMK measures only the minimum knowledge. Additional knowledge that could be important or relevant is not measured.
